# Phytoplasmas–The “Crouching Tiger” Threat of Australian Plant Pathology

**DOI:** 10.3389/fpls.2017.00599

**Published:** 2017-04-26

**Authors:** Jian Liu, David Gopurenko, Murray J. Fletcher, Anne C. Johnson, Geoff M. Gurr

**Affiliations:** ^1^State Key Laboratory of Ecological Pest Control for Fujian and Taiwan Crops, Fujian Agriculture and Forestry University Fuzhou, China; ^2^Institute of Applied Ecology, Fujian Agriculture & Forestry University Fuzhou, China; ^3^Graham Centre for Agricultural Innovation (Charles Sturt University & NSW Department of Primary Industries) Orange, NSW, Australia; ^4^NSW Department of Primary Industries, Wagga Wagga Agricultural Institute Wagga Wagga, NSW, Australia

**Keywords:** “*Candidatus* Phytoplasma”, 16S rRNA, biosecurity, taxonomy, biodiversity, vector, seed transmission, host range

## Abstract

Phytoplasmas are insect-vectored bacteria that cause disease in a wide range of plant species. The increasing availability of molecular DNA analyses, expertise and additional methods in recent years has led to a proliferation of discoveries of phytoplasma-plant host associations and in the numbers of taxonomic groupings for phytoplasmas. The widespread use of common names based on the diseases with which they are associated, as well as separate phenetic and taxonomic systems for classifying phytoplasmas based on variation at the 16S rRNA-encoding gene, complicates interpretation of the literature. We explore this issue and related trends through a focus on Australian pathosystems, providing the first comprehensive compilation of information for this continent, covering the phytoplasmas, host plants, vectors and diseases. Of the 33 16Sr groups reported internationally, only groups II, XI, XII, XXIII, XXV, and XXXIII have been recorded in Australia and this highlights the need for ongoing biosecurity measures to prevent the introduction of additional pathogen groups. Many of the phytoplasmas reported in Australia have not been sufficiently well studied to assign them to 16Sr groups so it is likely that unrecognized groups and sub-groups are present. Wide host plant ranges are apparent among well studied phytoplasmas, with multiple crop and non-crop species infected by some. Disease management is further complicated by the fact that putative vectors have been identified for few phytoplasmas, especially in Australia. Despite rapid progress in recent years using molecular approaches, phytoplasmas remain the least well studied group of plant pathogens, making them a “crouching tiger” disease threat.

## Introduction

Phytoplasmas are insect-vectored bacteria that cause disease in a wide range of plant species (Lee et al., 2000; IRPCM, 2004; Bertaccini et al., 2014; Marcone, 2014). They contrast with other phloem-limited bacteria (Gram-negative proteobacteria such as liberibacters and phlomobacters Bove and Garnier, 2003) which are vectored by the same types of insects, in lacking a cell wall and in having a much reduced genome size (0.53–1.2 kb; Streten and Gibb, 2006). Spiroplasmas, another group of insect vectored plant pathogenic microbes, share the absence of a cell wall but differ from phytoplasmas in that some are culturable *in vitro*. In this mini review we seek to raise awareness of the importance of this group of plant pathogens, summarizing five key issues that mean the threat they pose to agricultural, ornamental and natural vegetation is not fully appreciated, making them worthy of the “crouching tiger” description. We focus principally on the phytoplasmas of Australia, as a sub-set of the large and rapidly-expanding global literature. We consider the phytoplasmas, associated plant diseases, plant hosts and the putative insect vectors (Table 1). Work from locations other than Australia is mentioned where appropriate to provide context and show where the issues we identify are generic rather than Australian-specific as well as to point out opportunities that are open for future study of Australian phytoplasma pathosystems. The only other reviews of phytoplasmas in Australia are at least a decade old and confined to tropical (Wilson et al., 2001) and sub-tropical regions (Streten and Gibb, 2006) whereas the scope of the present review includes the agriculturally important temperate zone. Reviews of varying levels of comprehensiveness are available for other geographical areas: New Zealand (Veerakone et al., 2015), Latin America (Pérez-López et al., 2016), the Pacific region (Davis and Ruabete, 2010) and Southern Italy (Marcone, 2011). Reflecting the large body of literature now available on phytoplasmas, the only reviews with an international scope are confined to particular crops such as coconut palm (Gurr et al., 2016), date palm (Gurr et al., 2015), fruit trees (Adams et al., 2001) and sugar cane (Smith et al., 2001).

**Table 1:** Taxonomic and biological information on phytoplasmas in Australia (empty cells denote the absence of available information).

**16Sr group**	***“Candidatus* Phytoplasma” name**	**Phytoplasma trivial name**	**Host plant species**	**Potential vectors**	**Location^+^**	References^*^
II	australasiae^a^	Australian lucerne yellows	*Medicago sativa, Carica papaya*	*Orosius argentatus, Austroagallia torrida, Orosius* spp., *Batracomorphus* sp.	South Australia, New South Wales, Northern Territory	Padovan and Gibb, 2001^*^; Pilkington et al., 2003^*^; Yang et al., 2013
II		Bonamia pannosa little leaf	*Bonamia pannosa*		Northern Territory	Schneider et al., 1999; Padovan and Gibb, 2001
II		Cactus witches' broom	*Carica papaya*		Northern Territory	Padovan and Gibb, 2001
II		Cocky apple witches' broom	*Planchonia careya*		Queensland	Davis et al., 2001
II		Waltheria little leaf	*Mitracarpus hirtus, Saccharum* sp*., Spermacocci sp., Waltheria indica, Carica papaya*		Northern Territory	Schneider et al., 1999; Tran-Nguyen et al., 2000; Padovan and Gibb, 2001; Wilson et al., 2001
II	australasiae	Tomato big bud	*Achyranthes aspera, Aeschynomene* spp.*, Alysicarpus rugosus, Amaranthus* sp., *Apium graveolens, Arachis* spp.*, Boeharvia* sp., *Brugmansia x candida, Capsicum annuum, Carica papaya, Catharanthus roseus, Cajanus cajan, Citrus paradisi, Crotalaria* spp., *Cenchrus ciliaris, Cichorium intybus, Cleome viscosa, Cucurbita maxima, Cynodon dactylon, Daucus carota, Emilia sonchifolia, Eragrostis falcata, Eriachne obtusa, Euphorbia milii, Evolvulus* sp., *Gerbera* sp., *Goodenia* sp., *Guizotia abyssinica, Ipomoea* spp.*, Lactuca sativa, Lycopersicon esculentum, Macroptilium* spp., *Medicago sativa, Mucuna pruriens, Passiflora* sp., *Phlox* sp., *Physalis minima, Ptilotus distans, Rhynchosia minima, Saccharum* sp., *Sarcochilus hartmanii × S. falcatus, Sesamum indicum, Sida cordifolia, Lycopersicon esculentum, Solanum melongena, Stylosanthes scabra, Trifolium repens, Vigna* spp.*, Vitis vinifera, Zinnia elegans*,	*Austroagallia torrida*	Northern Territory, New South Wales, Queensland, Western Australia, Victoria	Gibb et al., 1995; Davis et al., 1997b; Gowanlock et al., 1998; De La Rue et al., 1999; Tran-Nguyen et al., 2000, 2003; Wilson et al., 2001^*^; Pilkington et al., 2004; Streten and Gibb, 2006
II	aurantifolia	Chickpea little leaf	*Cicer arietinum*		Western Australia	Saqib et al., 2005
II	australasiae	Papaya yellow crinkle	*Carica papaya*		Queensland	Gibb et al., 1996; White et al., 1998
II	australasiae	Papaya mosaic	*Carica papaya*		Queensland	Gibb et al., 1996; White et al., 1998
II		Tree medic witches' broom	*Medicago arborea*		South Australia	Yang et al., 2013
II		Pigeonpea phyllody	*Cajanus cajan*		South Australia	Yang et al., 2013
II		Pigeon pea little leaf	*Arachis* spp., *Catharanthus roseus, Crotalaria* spp.*, Desmodium triflorum, Indigofera* sp., *Macroptilium bracteatum Pterocaulon* sp. *Sesuvium portulacastrum, Stylosanthes* spp.*, Vigna radiata*		Northern Territory, Queensland, Torres Strait	Schneider et al., 1999; De La Rue et al., 2001; Padovan and Gibb, 2001; Wilson et al., 2001; Davis et al., 2003; Streten and Gibb, 2006
II-D	australasiae	Pale purple coneflower witches' broom	*Echinacea pallida*		Tasmania	Pearce et al., 2011
II-D	australasiae	Sweet potato little leaf	*Alysicarpus vaginalis, Aphyllodium* sp., *Arachis* spp., *Cajanus marmoratus, Carica papaya, Catharanthus roseus, Centrosema pascuorum, Citrus* sp., *Cleome viscosa, Crotalaria* spp., *Cucurbita maxima, Cyanthillium* spp., *Desmodium* spp., *Emilia sonchifolia, Indigofera* spp., *Ipomoea batatas, Macroptilium gracile, Medicago sativa, Mitracarpus hirtus, Nicotiana tabacum, Pachyrhizus erosus, Physalis minima, Rhynchosia minima, Senna obtusifolia, Sesamum indicum, Stylosanthes* ssp., *Tridax procumbens, Vigna* spp.	*Austroagallia torrida, Orosius* spp., *Batracomorphus* sp.	Torres Strait, Northern Territory, Western Australia, New South Wales	Gibb et al., 1995^*^; Liu et al., 1996; Davis et al., 1997b; Schneider and Gibb, 1997^*^; De La Rue et al., 1999, 2001; Padovan and Gibb, 2001; Wilson et al., 2001; Davis et al., 2003; Streten and Gibb, 2006; Tairo et al., 2006; Tran-Nguyen et al., 2012
XI-B		Cynodon white leaf	*Cynodon dactylon, Dactyloctenium aegyptium*		Northern Territory, Western Australia	Schneider et al., 1999; Tran-Nguyen et al., 2000; Blanche et al., 2003
XI-B		Sorghum grassy shoot	*Dactyloctenium* spp.*, Sorghum stipoideum, Whiteochloa* spp., *Chloris inflata, Whiteochloa cymbiformis*		Western Australia, Northern Territory	Tran-Nguyen et al., 2000; Blanche et al., 2003
XII		Australian lucerne yellows	*Medicago sativa*		New South Wales	Getachew et al., 2007
XII		Papaya dieback	*Carica papaya*		Queensland	Gibb et al., 1996; White et al., 1998
XII-B	australiense	Pumpkin yellow leaf curl	*Cucurbita maxima, C. moschata*		Queensland, Western Australia, Northern Territory	Streten et al., 2005
XII-B	australiense	Cenchrus bunchy shoot	*Cenchrus setiger*		Western Australia	Tran-Nguyen et al., 2000
XII-B	australiense	Strawberry green petal disease	*Fragaria* x *ananassa*		Queensland	Padovan et al., 2000
XII-B	australiense	Strawberry lethal yellows	*Fragaria* x *ananassa*		Queensland	Padovan et al., 2000
XII-B	australiense	Australian grapevine yellows^b^	*Vitis vinifera, Carica papaya*		South Australia, Queensland	Davis et al., 1997a,b; Davis and Sinclair, 1998; Davis et al., 2003
XXIII^c^		Buckland Valley grapevine yellows	*Vitis vinifera*		Victoria	Constable et al., 2003; Streten and Gibb, 2006; Zhao and Davis, 2016
XXV^d^		Weeping tea tree witches' broom	*Melaleuca* spp.		Queensland	Davis et al., 2003; Zhao and Davis, 2016
XXXIII		Allocasuarina yellows	*Allocasuarina muelleriana*		South Australia	Gibb et al., 2003; Zhao and Davis, 2016
		Poinsettia branching^e^	*Euphorbia pulcherrima*			Schneider et al., 1999
		Galactia little leaf	*Galactia tenuiflora*		Northern Territory	Schneider et al., 1999; Padovan and Gibb, 2001
		Sorghum bunchy shoot	*Sorghum stipoideum*			Tran-Nguyen et al., 2000
		Stylosanthes little leaf	*Arachis pintoi, Carica papaya, Saccharum* sp*., Sesuvium portulacastrum, Stylosanthes scabra*	*Austroagallia torrida, Orosius* spp., *Batracomorphus* sp.	Northern Territory, Queensland, New South Wales	Schneider et al., 1999; Tran-Nguyen et al., 2000; De La Rue et al., 2001; Padovan and Gibb, 2001; Davis et al., 2003; Gopurenko et al., 2016
		Sugarcane white leaf	*Saccharum* sp.		Western Australia, Queensland	Tran-Nguyen et al., 2000
		Vigna little leaf	*Vigna lanceolata, Carica papaya, Tridax procumbens*	*Austroagallia torrida, Batracomorphus* sp.	Northern Australia	Schneider et al., 1999; De La Rue et al., 2001; Padovan and Gibb, 2001
		Mundulla yellows disease^f^	*Eucalyptus camaldulensis, E. baxteri, E. leucoxylon*		South Australia	Hanold et al., 2006
		Paulownia witches' broom^g^	*Paulownia* sp.		Western Australia	Bayliss et al., 2005

* *Denotes reference for vector data*.

+ *Location data are from the listed references but not every plant species was diseased in every location*.

a *A new taxon, Ca. Phytoplasma australasia was proposed (White et al., 1998) to include the phytoplasma associated with papaya yellow crinkle and papaya mosaic (as well as tomato big bud) but later revised to “Ca. australasiae” (to include the papaya-associated phytoplasmas but not TBB; Firrao et al., 2005)*.

b *Davis and Sinclair (1998) moved the AGY phytoplasma from the 16SrI group into the stolbur group (16SrXII) and designated it subgroup B*.

c *Constable et al. (2003) reported a close relationship to 16Sr I. Zhao and Davis (2016) subsequently placed this into a new group: 16SrXXIII*.

d *Zhao and Davis (2016) placed this into this new group and potentially a new “Ca. Phytoplasma” species*.

e *This phytoplasma has not been found in economically important field crops*.

f *Tentative data only for a phytoplasma etiology*.

g RFLP patterns showed high similarity to “Candidatus Phytoplasma australiense.”

## Issue 1: phytoplasmology is a young science

Difficulties in studying phytoplasmas greatly limited early progress because phytoplasmas cannot be grown in axenic culture. Researchers were reliant initially on symptomology and transmission experiments, sometimes using grafting or the parasitic plant dodder (*Cuscuta* spp.) as in Australian work by Gibb et al. (1995), to study symptoms and host ranges but were unable to determine the nature of the pathogen or differentiate phytoplasmas from plant pathogenic viruses. Electron microscopy allowed phytoplasma bodies to be visualized in plant and insect vector tissue and differentiation of phytoplasmas from viruses. Bertaccini and Duduk (2010) provide a useful summary of the development of methods in phytoplasmology. Enzyme-linked immunosorbent assay (ELISA)-based methods began to emerge in the 1980s allowing more rapid detection and identification. The development of polymerase chain reaction (PCR) and restriction fragment length polymorphism (RFLP) methods for the detection and identification of phytoplasmas since the early 1990s allowed major advances, particularly in diagnostics and development of a genetic system for phenetic group classifications of phytoplasmas. By 1998, the international total of 34 representative phytoplasma strains were differentiated into 14 groups and 32 sub-groups based on similarity coefficients derived from RFLP analyses (Lee et al., 1998; Duduk and Bertaccini, 2011). More recent work has extended these counts to 33 groups and at least 100 sub-groups (Dickinson and Hodgetts, 2013; Davis et al., 2015; Zhao and Davis, 2016).

The increasingly widespread availability of molecular methods, equipment and expertise in recent decades has led to a proliferation of discoveries of phytoplasma-plant host associations and in taxonomic groupings for phytoplasmas. Many articles on phytoplasma pathosystems published this century are “first report” or “first record” articles for phytoplasmas in geographical regions or report known phytoplasmas from new host plant species. More fundamentally, new taxa of phytoplasma are being reported on a frequent basis. Progress has accelerated with the development of new approaches for detection and study of phytoplasmas. A prominent example is loop-mediated isothermal amplification (LAMP; Obura et al., 2011; Dickinson, 2015). This approach offers the advantages of low cost and high sensitivity, low risk of cross-contamination because reaction vessels do not need to be opened and, most especially, use in a kit form that requires very little training and can be used in the field (Hodgetts et al., 2011; Dickinson, 2015; Kogovšek et al., 2015). Though LAMP assays have yet to be used in studies of Australian phytoplasma pathosystems they have recently proven useful in studies of Bogia coconut syndrome in nearby Papua New Guinea (Lu et al., 2016).

In parallel to the use of simple LAMP diagnostics, next-generation sequencing (NGS) technologies have provided genomic characterization of phytoplasmas, and this has profoundly advanced the understanding of phytoplasma evolution and pathogenicity over recent years (Marcone, 2014). To date, complete genomes of six phytoplasma strains (Oshima et al., 2004; Bai et al., 2006; Kube et al., 2008; Tran-Nguyen et al., 2008; Andersen et al., 2013; Orlovskis et al., 2017) and an additional 16 draft or incomplete genomes have been reported (refer Genomes OnLine database [GOLD]; https://gold.jgi.doe.gov).

Comparative genomic analyses of phytoplasma and other mollicutes have provided direct evidence of reduced genomic complexity in phytoplasmas, characterized by encoding for fewer metabolic functions and pathways (Oshima et al., 2004). This highlights the obligate adaptation to, and reliance on, varied metabolic substrates available in hosts and vectors by phytoplasmas (Kube et al., 2013). Genomic studies have also detailed genes putatively encoding for phytoplasma virulence factors, including “effector” proteins which phytoplasmas produce and secrete to alter host cell activities, thereby modifying host development and reducing host defences against herbivorous arthropod vectors (Hogenhout and Loria, 2008; Hogenhout et al., 2008, 2009). Interestingly, comparative genomic analysis of five diverse phytoplasma groups failed to detect a consistent shared set of predicted secreted effector encoding genes (Wang et al., 2014). This suggests virulence encoding genes are likely to be diverse among phytoplasma strains, and may explain the wide range of pathogenicity in different 16Sr groups. Other work (Chung et al., 2013) indicates that horizontal gene flow of mobile genetic elements among some divergent phytoplasma strains may have facilitated horizontal transfer of effector genes linked to the mobile elements, adding a novel pathway for increasing the adaptive potential of phytoplasmas with regards to their hosts. NGS methods have been applied also for population metagenomics, allowing valuable insight into the ecology and dynamics of phytoplasmas and their relationships to hosts (Nicolaisen et al., 2011). Finally, phylogenetic analyses of phytoplasmas using NGS data are likely to improve understanding of systematic and taxonomic relationships in the genus, traditionally reliant on analyses of the 16S rRNA gene region (see Issue 3) and (in some cases) use of additional informative loci for comparison of very recently diverged strains (Al-Abadi et al., 2016).

Anticipated widespread use of affordable NGS services will result in a proliferation of the availability of published phytoplasma genomes and meta-genomic analyses, and this will ultimately lead to more advanced understanding of evolutionary relationships of species in this genus, and their interactive pathways with hosts and vectors including for Australian pathosystems.

## Issue 2: complex taxonomic nomenclature

A major impediment to comprehension of the phytoplasma literature and comparisons between studies, particularly for the non-specialist, is the taxonomic nomenclature with three systems currently in use. First, reflecting the history of phytoplasmology described above, the early literature uses disease common names based on symptoms (e.g., little leaf, yellows, witches' broom) often coupled with the host plant's name. These disease common names have been applied also to the phytoplasmas and continue to be used frequently in recent literature. Examples from the Australian literature include Buckland Valley grapevine yellows phytoplasma, tomato big bud phytoplasma and Cockey apple witches' broom phytoplasma (Table 1). This allows great scope for confusion because a given phytoplasma can be found in multiple plant species and can cause different disease symptoms in different hosts.

Second, as molecular methods became available, workers were able to group and phenetically classify phytoplasmas using restricted fragment length polymorphism (RFLP) analysis of a PCR amplified portion of the 16S rRNA gene with a defined set of restriction enzymes (Lee et al., 1998). The RFLP profiles generated for different phytoplasmas are generally consistent with sequence-based phylogenetic analyses of the 16S rRNA gene, particularly in the co-identification and grouping of related strains. The 33 16Sr groups currently defined each have a similarity of <85% compared with any representative phytoplasma from within an established 16Sr group (Zhao and Davis, 2016). Table 1 summarizes available information on the 16Sr groups reported in Australian studies. Of the 33 16Sr groups reported internationally, only groups II, XI, XII, XXIII, XXV, and XXXIII have been recorded in Australia and this highlights the need for ongoing biosecurity measures to prevent the introduction of additional pathogen groups.

Third, phytoplasmas are classified in the provisional genus “*Candidatus* Phytoplasma” (IRPCM, 2004). To date, there are 42 formally described species and ten potentially novel phytoplasma species (Davis et al., 2015). This number exceeds the current number of 16s rRNA groups because some of these groups contain several “*Candidatus* Phytoplasma” species. At least 100 subgroups are known (Dickinson and Hodgetts, 2013). According to Phytoplasma/Spiroplasma Working Team-Phytoplasma Taxonomy Group, a novel “*Ca*. Phytoplasma” species description should refer to a single, unique 16S rRNA gene sequence (>1,200 bp), and a strain can be recognized as a novel “*Ca*. Phytoplasma” species if its 16S rRNA gene sequence has <97.5% similarity to that of any previously described “*Ca*. Phytoplasma” species (Duduk and Bertaccini, 2011). Additional biological characters such as antibody specificity, host range and vector transmission specificity as well as genetic markers can also be used in an integrative taxonomy approach for species differentiation. Of the 42 recognized “*Ca*. Phytoplasma” species, only *Ca*. Phytoplasma aurantifolia, *Ca*. Phytoplasma australasiae and *Ca*. Phytoplasma australiense are reported in Australia (Table 1) but uncertainty exists because many papers appear without *Ca*. Phytoplasma names which are used consistently only in the case of the GenBank database.

The general literature uses a mix of *Ca*. Phytoplasma names, 16Sr group and sub-group names, and common names that often reflect the host plant or symptom (Table 1). Though formally named *Ca*. Phytoplasma species each align with a group or sub group in the 16Sr system, many groups and sub-groups do not currently have a “*Candidatus* Phytoplasma” species name.

## Issue 3: large and poorly understood biodiversity among pathogens and wide host ranges

Whilst it is important to note that a given type of phytoplasma can attack more than one plant species, and that a given plant species can be attacked by multiple types of phytoplasma, the list of phytoplasma disease common names from Australia (Table 1) is instructive in indicating the wide range of plant species that are affected, even for specific phytoplasmas. Examples extend over forage, broadacre and horticultural crops of a perennial and annual nature, as well as ornamental and uncultivated (i.e., natural vegetation) species.

There is an especially poor knowledge of phytoplasma pathosystems in temperate Australia, largely because of the tropical and sub-tropical zone focus of most of the key Australian workers in the last 20 years. Many of the phytoplasmas reported in Australia have not been sufficiently well studied to assign them to 16Sr groups so it is likely that unrecognized groups—as well as sub-groups—are present, particularly in native vegetation.

Great efforts are currently being made to define the extent and diversity of phytoplasmas in Australia (most recently the discovery of Stylosanthes little leaf 16Sr XII phytoplasma from lucerne in New South Wales Gopurenko et al., 2016) and elsewhere but this remains a challenge because of the apparent high levels of biological diversity. As we approach a definitive list of 16Sr groups and sub groups, “*Ca*. phytoplasma” species names need to be assigned that avoid scope for confusion over the host plant, perhaps by wider use of names that reflect the country in which the phytoplasma was first discovered (as is the case for *Ca*. Phytoplasma australiense, though noting the potential confusion with *Ca*. Phytoplasma australasiae and the redundant *Ca*. Phytoplasma australasia Table 1) or a more neutral name based, perhaps, on the discoverer rather than the use of host plant taxon names such as pini, palmicola, pruni and oryzae, all of which are reported internationally though not from Australia.

Reflecting and compounding the three, related issues outlined above, there is no universally recognized taxonomic resource for phytoplasmas. For other higher taxa, there is a name-bearing type specimen lodged in an accessible scientific collection that can be checked by subsequent researchers, allowing comparison with unknown or new taxa. With phytoplasmas, however, the extreme difficulties associated with axenic culture mean that the “type” is a DNA sequence in GenBank®, the National Institutes of Health's annotated collection of all publicly available DNA sequences (Benson et al., 2013), and not a biological specimen. Further, there is no common platform for registering phytoplasma groups and sub-groups (Zhao and Davis, 2016). The *International Journal of Systematics and Evolution* (formerly the *International Journal of Systematic Bacteriology*) is the official journal of record for novel prokaryotic taxa since it is the official publication of the International Committee on Systematics of Prokaryotes and the Bacteriology and Applied Microbiology Division of the International Union of Microbiological Societies. Accordingly, new “*Ca*. Phytoplasma” species are published in this journal. Importantly, however, new 16Sr groups can be (and are) published in other journals provided that the group contains a previously described “*Ca*. Phytoplasma” species. This lack of a common platform for group names means that a given 16Sr group number can inadvertently be applied by different authors to different “*Ca*. Phytoplasma” taxa. Zhao and Davis (2016) provide the example that “*Ca*. Phytoplasma” allocasuarinae was assigned as 16SrXXXIII (Bertaccini et al., 2014) but the same 16Sr group name was also subsequently used for another new (Chilean grapevine phytoplasma) group (Pérez-López et al., 2016). This led to the recent suggestion (Zhao and Davis, 2016) that 16Sr groups and sub-groups should be registered via a web-based portal linked to iPhyClassifier, itself a web-based resource that allows identification and classification of phytoplasmas by simulating restriction enzyme digestions and electrophoresis to produce “virtual” RFLP patterns for submitted query sequences.

## Issue 4: challenges associated with vector studies

The insect vectors of phytoplasmas are yet to be investigated thoroughly in Australia and more generally and this is a major constraint on the development of disease management approaches that focus on vector control. The Australian literature provides putative vector names only for Australian lucerne yellows, sweetpotato little leaf, tomato big bud, Stylosanthes little leaf and Vigna little leaf phytoplasmas (Table 1).

Since phytoplasmas are phloem-limited pathogens, they are transmitted by phloem-feeding hemipteran insects, especially leafhoppers and planthoppers (Weintraub and Beanland, 2006) though psyllids are responsible in some non-Australian pathosystems (Carraro et al., 1998). The identification of potential vector species involves field surveys to determine Hemiptera species that are spatially and temporally associated with plant symptoms followed by the use of PCR to detect phytoplasma DNA in the insects. However, the detection of phytoplasma DNA in an insect does not establish vector status because DNA may be confined to the gut as a result of feeding on an infected host plant rather than the pathogen having colonized the salivary glands making it a competent vector (Vega et al., 1993; Danielli et al., 1996). Definitive proof of vector status can be obtained from transmission tests confining putative vectors on phytoplasma-free host plants in insect-proof cages. Whilst this has been done in a preliminary manner for Australian lucerne yellows (e.g., Pilkington et al., 2004), vector transmission testing is logistically demanding, especially if host plants are large perennial species, if the pathogen is vectored inefficiently or if symptoms develop slowly (Gurr et al., 2016). A relatively new method that is intermediate in ease of use and the level of proof involves holding individual insects in vessels from which they can feed on a sucrose solution through a parafilm barrier with subsequent PCR-based detection of phytoplasma DNA in the medium (Tanne et al., 2001). Though this approach involves many samples (i.e., one per insect rather than one per plant as in the case of a cage transmission test) a recent study has employed LAMP to readily handle the large numbers of samples associated with screening multiple putative vector species of Bogia coconut syndrome (Lu et al., 2016).

## Issue 5: possibility of seed transmission

Among the crop species of great economic importance in Australia, tomato, canola and maize have all been experimentally shown to exhibit seed transmission of phytoplasmas, a phenomenon previously considered unlikely and this adds to the importance of ongoing biosecurity and research efforts. In that study, PCR was used to detect phytoplasma DNA in laboratory grown seedlings arising from seed of phytoplasma infected plants of oilseed rape, tomato and corn (Calari et al., 2011). Caution is required in extrapolating from those tests with herbaceous annuals, however, because earlier reports of phytoplasma DNA in embryos of various woody perennial plant species including coconut have not been followed-up with conclusive evidence of seed transmissibility (Nipah et al., 2007) but see Oropeza et al. (2017).

## Conclusion and perspective

The ubiquity of PCR capacity and the advent of LAMP kits for simple diagnostics and NGS for advanced genomic and meta-genomic analyses will spur the discovery of many new phytoplasma diseases and allow rapid advances in understanding of phytoplasma biodiversity and biology in Australia and internationally. This has great practical relevance to address issues such as vector identity and the field-relevance of seed transmission. Because Australia is an island nation with well-established biosecurity measures, improvements in knowledge of phytoplasma biodiversity, host range and detection will be particularly valuable in the prevention of and appropriate responses to phytoplasma incursions. Further sustained research effort by specialists is important but this needs to be complemented by efforts to make this group of pathogens better known and easier to understand, especially taxonomically.

## Author contributions

All authors listed, have made substantial, direct and intellectual contribution to the work, and approved it for publication.

## Funding

This work was supported by a Graham Centre New Initiative Grant to the authors. GG is supported by a Chinese Government Thousand Talents Program fellowship (KX1452102).

### Conflict of interest statement

The authors declare that the research was conducted in the absence of any commercial or financial relationships that could be construed as a potential conflict of interest.

## References

[B1] Adams A. N. Davies D. L. Kirby M. J. (2001). Virus and phytoplasma detection in fruit trees. Outlook Agric. 30, 45–54. 10.5367/000000001101293454

[B2] Al-Abadi S. Y. Al-Sadi A. M. Dickinson M. Al-Hammadi M. S. Al-Shariqi R. Al-Yahyai A. (2016). Population genetic analysis reveals a low level of genetic diversity of “*Candidatus* Phytoplasma aurantifolia” causing witches' broom disease in lime. BMC Microbiol. 5:1701 10.1186/s40064-016-3401-0 PMC5047875 27757373

[B3] Andersen M. T. Liefting L. W. Havukkala I. Beever R. E. (2013). Comparison of the complete genome sequence of two closely related isolates of “*Candidatus* Phytoplasma australiense” reveals genome plasticity. BMC Genomics 14:529. 10.1186/1471-2164-14-529 23915186 PMC3750655

[B4] Bai X. Zhang J. Ewing E. Miller S. A. Radek A. Schevchenko D.. (2006). Living with genome instability: the adaptation of phytoplasmas to diverse environments of their insect and plant hosts. J. Bacteriol. 188, 3682–3696. 10.1128/JB.188.10.3682-3696.2006 16672622 PMC1482866

[B5] Bayliss K. L. Saqib M. Dell B. Jones M. G. K. Hardy G. E. S. J. (2005). First record of “*Candidatus* Phytoplasma australiense” in *Paulownia* trees. Australas. Plant Pathol. 34, 123–124. 10.1071/AP04089

[B6] Benson D. A. Cavanaugh M. Clark K. Karsch-Mizrachi I. Lipman D. J. Ostell J.. (2013). GenBank. Nucleic Acids Res. 41, D36–D42. 10.1093/nar/gks1195 23193287 PMC3531190

[B7] Bertaccini A. Duduk B. (2010). Phytoplasma and phytoplasma diseases: a review of recent research. Phytopathol. Mediterr. 48, 355–378. 10.14601/Phytopathol_Mediterr-3300

[B8] Bertaccini A. Duduk B. Paltrinieri S. Contaldo N. (2014). Phytoplasmas and phytoplasma diseases: a severe threat to agriculture. Am. J. Plant Sci. 5, 1763–1788. 10.4236/ajps.2014.512191

[B9] Blanche K. R. Tran-Nguyen L. T. T. Gibb K. S. (2003). Detection, identification and significance of phytoplasmas in grasses in northern Australia. Plant Pathol. 52, 505–512. 10.1046/j.1365-3059.2003.00871.x

[B10] Bove J. Garnier M. (2003). Phloem-and xylem-restricted plant pathogenic bacteria. Plant Sci. 164, 423–438. 10.1016/S0168-9452(03)00033-5

[B11] Calari A. Paltrinieri S. Contaldo N. Sakalieva D. Mori N. Duduk B. (2011). Molecular evidence of phytoplasmas in winter oilseed rape, tomato and corn seedlings. Bull. Insectol. 64, S157–S158.

[B12] Carraro L. Osler R. Loi N. Ermacora P. Refatti E. (1998). Transmission of European stone fruit yellows phytoplasma by *Cacopsylla pruni*. J. Plant Pathol. 80, 233–239.

[B13] Chung W.-C. Chen L.-L. Lo W.-S. Lin C.-P. Kuo C.-H. (2013). Comparative analysis of the peanut witches'-broom phytoplasma genome reveals horizontal transfer of potential mobile units and effectors. Robinson-Rechavi M. ed. PLoS ONE 8:e62770. 10.1371/journal.pone.0062770 23626855 PMC3633829

[B14] Constable F. E. Whiting J. R. Jones J. Gibb K. S. Symons R. H. (2003). The distribution of grapevine yellows disease associated with the buckland valley grapevine yellows phytoplasma. J. Phytopathol. 151, 65–73. 10.1046/j.1439-0434.2003.00681.x

[B15] Danielli A. Bertaccini A. Vibio M. Mori N. Murari E. Posenato G. (1996). Detection and molecular characterization of phytoplasmas in the planthopper *Metcalfa pruinosa* (Say) (Homoptera: Flatidae). Phytopathol. Mediterr. 35, 62–65.

[B16] Davis R. Dally E. L. Gundersen D. E. Lee I. M. Habili N. (1997a). “*Candidatus* Phytoplasma australiense,” a New Phytoplasma Taxon Associated with Australian Grapevine Yellows. Int. J. Syst. Evol. Microbiol. 47, 262–269. 10.1099/00207713-47-2-262 9103609

[B17] Davis R. E. Sinclair W. A. (1998). Phytoplasma identity and disease etiology. Phytopathology 88, 1372–1376. 10.1094/PHYTO.1998.88.12.1372 18944842

[B18] Davis R. I. Henderson J. Jones L. M. McTaggart A. R. O'Dwyer C. Tsatsia F. (2015). First record of a wilt disease of banana plants associated with phytoplasmas in Solomon Islands. Australas. Plant Dis. Notes 10:14 10.1007/s13314-015-0163-4

[B19] Davis R. I. Jacobson S. C. Rue S. J. Tran-Nguyen L. Gunua T. G. Rahamma S. (2003). Phytoplasma disease surveys in the extreme north of Queensland, Australia, and the island of New Guinea. Australas. Plant Pathol. 32, 269–277. 10.1071/AP03020

[B20] Davis R. I. Jacobson S. C. Waldeck G. J. De La Rue S. J. Gibb K. S. (2001). A witches' broom of cocky apple (*Planchonia careya*) in north Queensland. Australas. Plant Pathol. 30, 179–179. 10.1071/AP01016

[B21] Davis R. I. Ruabete T. K. (2010). Records of plant pathogenic viruses and virus-like agents from 22 Pacific island countries and territories: a review and an update. Australas. Plant Pathol. 39, 265–291. 10.1071/AP10047

[B22] Davis R. Schneider B. Gibb K. (1997b). Detection and differentiation of phytoplasmas in Australia. Aust. J. Agric. Res. 48, 535–544. 10.1071/A96114

[B23] De La Rue S. J. Schneider B. Gibb K. S. (1999). Genetic variability in phytoplasmas associated with papaya yellow crinkle and papaya mosaic diseases in Queensland and the Northern Territory. Australas. Plant Pathol. 28, 108–114. 10.1071/AP99019

[B24] De La Rue S. Padovan A. Gibb K. (2001). *Stylosanthes* is a Host for Several Phytoplasmas, One of which Shows Unique 16S-23S Intergenic Spacer Region Heterogeneity. J. Phytopathol. 149, 613–619. 10.1046/j.1439-0434.2001.00683.x

[B25] Dickinson M. (2015). Loop-mediated isothermal amplification (LAMP) for detection of phytoplasmas in the field, in Plant Pathology, ed Lacomme C.(New York, NY: Springer), 99–111 10.1007/978-1-4939-2620-6_8 25981249

[B26] Dickinson M. Hodgetts J. (eds) (2013). Phytoplasma: Methods and Protocols, Dordrecht. Netherlands: Science+Business Media.

[B27] Duduk B. Bertaccini A. (2011). Phytoplasma classification: taxonomy based on 16S ribosomal gene, is it enough? Phytopathogenic Mollicutes 1, 3–13. 10.5958/j.2249-4669.1.1.001

[B28] Firrao G. Gibb K. Streten C. (2005). Short taxonomic guide to the genus “*Candidatus* Phytoplasma.” J. Plant Pathol. 87, 249–263. 10.4454/jpp.v87i4.926

[B29] Getachew M. A. Mitchell A. Gurr G. M. Fletcher M. J. Pilkington L. J. Nikandrow A. (2007). First report of a “*Candidatus* phytoplasma australiense”-related strain in lucerne (*Medicago sativa*) in Australia. Plant Dis. 91, 111–111. 10.1094/PD-91-0111A 30781081

[B30] Gibb K. Padovan A. Mogen B. (1995). Studies on sweet potato little-leaf phytoplasma detected in sweet potato and other plant species growing in Northern Australia. Phytopathology 85, 169–174. 10.1094/Phyto-85-169

[B31] Gibb K. Persley D. Schneider B. Thomas J. (1996). Phytoplasmas associated with papaya diseases in Australia. Plant Dis. 80, 174–178. 10.1094/PD-80-0174

[B32] Gibb K. S. Tran-Nguyen L. T. T. Randles J. W. (2003). A new phytoplasma detected in the South Australian native perennial shrub, *Allocasuarina muelleriana*. Ann. Appl. Biol. 142, 357–364. 10.1111/j.1744-7348.2003.tb00261.x

[B33] Gopurenko D. Fletcher M. J. Liu J. Gurr G. M. (2016). Expanding and exploring the diversity of phytoplasmas from lucerne (*Medicago sativa*). Sci. Rep. 6:37746. 10.1038/srep37746 27886229 PMC5123570

[B34] Gowanlock D. H. Ogle H. J. Gibb K. S. (1998). Phytoplasma associated with virescence in an epiphytic orchid in Australia. Australas. Plant Pathol. 27, 265–268. 10.1071/AP98031

[B35] Gurr G. M. Bertaccini A. Gopurenko D. Krueger R. R. Alhudaib K. A. Liu J. (2015). Phytoplasmas and their insect vectors: implications for date palm, in Sustainable Pest Management in Date Palm: Current Status and Emerging Challenges, eds Wakil W. Romeno Faleiro J. Miller A. T.(Cham: Springer International Publishing), 287–314.

[B36] Gurr G. M. Johnson A. Ash G. Wilson B. Ero M. Pilotti C.. (2016). Coconut lethal yellowing diseases: a phytoplasma threat to palms of global economic and social significance. Front. Plant Sci. 7:5121. 10.3389/fpls.2016.01521 27833616 PMC5080360

[B37] Hanold D. Gowanlock D. Stukely M. J. C. Habili N. Randles J. W. (2006). Mundulla Yellows disease of eucalypts: descriptors and preliminary studies on distribution and etiology. Australas. Plant Pathol. 35, 199–215. 10.1071/AP06013

[B38] Hodgetts J. Tomlinson J. Boonham N. González-Martín I. Nikolić P. Swarbrick P. (2011). Development of rapid in-field loop-mediated isothermal amplification (LAMP) assays for phytoplasmas. Bull. Insectol. 64, S41–S42.

[B39] Hogenhout S. A. Loria R. (2008). Virulence mechanisms of Gram-positive plant pathogenic bacteria. Curr. Opin. Plant Biol. 11, 449–456. 10.1111/j.1364-3703.2008.00472.x 18639483

[B40] Hogenhout S. A. Oshima K. Ammar E.-D. Kakizawa S. Kingdom H. N. Namba S. (2008). Phytoplasmas: Bacteria that manipulate plants and insects. Mol. Plant Pathol. 9, 403–423. 10.1111/j.1364-3703.2008.00472.x 18705857 PMC6640453

[B41] Hogenhout S. A. Van der Hoorn R. A. L. Terauchi R. Kamoun S. (2009). Emerging concepts in effector biology of plant-associated organisms. Mol. Plant Microbe Interact. 22, 115–22. 10.1111/j.1364-3703.2008.00472.x 19132864

[B42] IRPCM (2004). “*Candidatus* Phytoplasma,” a taxon for the wall-less, non-helical prokaryotes that colonize plant phloem and insects. Int. J. Syst. Evol. Microbiol. 54, 1243–1255. 10.1099/ijs.0.02854-0 15280299

[B43] Kogovšek P. Hodgetts J. Hall J. Prezelj N. Nikolić P. Mehle N.. (2015). LAMP assay and rapid sample preparation method for on-site detection of flavescence dorée phytoplasma in grapevine. Plant Pathol. 64, 286–296. 10.1111/ppa.12266 26146413 PMC4480326

[B44] Kube M. Schneider B. Kuhl H. Dandekar T. Heitmann K. Migdoll A. M.. (2008). The linear chromosome of the plant-pathogenic mycoplasma ‘*Candidatus* Phytoplasma mali’. BMC Genomics 9:306. 10.1186/1471-2164-9-306 18582369 PMC2459194

[B45] Kube M. Siewert C. Migdoll A. M. Duduk B. Holz S. Rabus R.. (2013). Analysis of the complete genomes of *Acholeplasma brassicae, A. palmae* and *A. laidlawii* and their comparison to the obligate parasites from “*Candidatus* Phytoplasma.” J. Mol. Microbiol. Biotechnol. 24, 19–36. 10.1159/000354322 24158107

[B46] Lee I.-M. Davis R. E. Gundersen-Rindal D. E. (2000). Phytoplasma: phytopathogenic mollicutes. Annu. Rev. Microbiol. 54, 221–255. 10.1146/annurev.micro.54.1.221 11018129

[B47] Lee I.-M. Gundersen-Rindal D. E. Davis R. E. Bartoszyk I. M. (1998). Revised classification scheme of phytoplasmas based on RFLP Analyses of 16S rRNA and ribosomal protein gene sequences. Int. J. Syst. Bacteriol. 48, 1153–1169. 10.1099/00207713-48-4-1153 9542097

[B48] Liu B. White D. Walsh K. Scott P. (1996). Detection of phytoplasmas in dieback, yellow crinkle, and mosaic diseases of papaya using polymerase chain reaction techniques. Aust. J. Agric. Res. 47, 387–394. 10.1071/AR9960387

[B49] Lu H. Wilson B. A. L. Ash G. J. Woruba S. B. Fletcher M. J. You M.. (2016). Determining putative vectors of the Bogia Coconut Syndrome phytoplasma using loop-mediated isothermal amplification of single-insect feeding media. Sci. Rep. 6:35801. 10.1038/srep35801 27786249 PMC5082361

[B50] Marcone C. (2011). Current status of phytoplasma diseases of medicinal and nutraceutical plants in Southern Italy. Bull. Insectol. 64, S233–S234.

[B51] Marcone C. (2014). Molecular biology and pathogenicity of phytoplasmas. Annals of Applied Biology, 165, 199–221. 10.1111/aab.12151

[B52] Nicolaisen M. Contaldo N. Makarova O. Paltrinieri S. Bertaccini A. (2011). Deep amplicon sequencing reveals mixed phytoplasma infection within single grapevine plants. Bull. Insectol. 64, S35–S36.

[B53] Nipah J. O. Jones P. Hodgetts J. Dickinson M. (2007). Detection of phytoplasma DNA in embryos from coconut palms in Ghana, and kernels from maize in Peru. Bull. Insectol. 60, 385–386.

[B54] Obura E. Masiga D. Wachira F. Gurja B. Khan Z. R. (2011). Detection of phytoplasma by loop-mediated isothermal amplification of DNA (LAMP). J. Microbiol. Methods 84, 312–316. 10.1016/j.mimet.2010.12.011 21185882

[B55] Orlovskis Z. Canale M. C. Haryono M. Lopes J. R. S. Kuo C.-H. Hogenhout S. A. (2017). A few sequence polymorphisms among isolates of Maize bushy stunt phytoplasma associate with organ proliferation symptoms of infected maize plants. Ann. Bot. 119, 869–884. 10.1093/aob/mcw213 28069632 PMC5379588

[B56] Oropeza C. Cordova I. Puch-Hau C. Castillo R. Chan J. L. Sáenz L. (2017). Detection of lethal yellowing phytoplasma in coconut plantlets obtained through *in vitro* germination of zygotic embryos from the seeds of infected palms. Ann. Appl. Biol. 10.1111/aab.12351. [Epub ahead of print].

[B57] Oshima K. Kakizawa S. Nishigawa H. Jung H. Y. Wei W. Suzuki S.. (2004). Reductive evolution suggested from the complete genome sequence of a plant-pathogenic phytoplasma. Nature Genet. 36, 27–29. 10.1038/ng1277 14661021

[B58] Padovan A. C. Gibb K. S. (2001). Epidemiology of phytoplasma diseases in papaya in Northern Australia. J. Phytopathol. 149, 649–658. 10.1046/j.1439-0434.2001.00688.x

[B59] Padovan A. Gibb K. Persley D. (2000). Association of “*Candidatus* Phytoplasma australiense” with green petal and lethal yellows diseases in strawberry. Plant Pathol. 49, 362–369. 10.1046/j.1365-3059.2000.00461.x

[B60] Pearce T. L. Scott J. B. Pethybridge S. J. (2011). First report of a 16SrII-D subgroup phytoplasma associated with pale purple coneflower Witches'-Broom disease in Australia. Plant Dis. 95, 773–773. 10.1094/PDIS-03-11-0155 30731927

[B61] Pérez-López E. Luna-Rodríguez M. Olivier C. Y. Dumonceaux T. J. (2016). The underestimated diversity of phytoplasmas in Latin America. Int. J. Syst. Evol. Microbiol. 66, 492–513. 10.1099/ijsem.0.000726 26519050

[B62] Pilkington L. J. Gibb K. S. Gurr G. M. Fletcher M. J. Nikandrow A. Elliott E. (2003). Detection and identification of a phytoplasma from lucerne with Australian lucerne yellows disease. Plant Pathol. 52, 754–762. 10.1111/j.1365-3059.2003.00934.x

[B63] Pilkington L. J. Gurr G. M. Fletcher M. J. Nikandrow A. Elliott E. (2004). Vector status of three leafhopper species for Australian lucerne yellows phytoplasma. Aust. J. Entomol. 43, 366–373. 10.1111/j.1440-6055.2004.00419.x

[B64] Saqib M. Bayliss K. L. Dell B. Hardf G. E. S. Jones M. G. K. (2005). First record of a phytoplasma-associated disease of chickpea (*Cicer arietinum*) in Australia. Australas. Plant Pathol. 34, 425–426. 10.1071/AP05047

[B65] Schneider B. Gibb K. S. (1997). Detection of phytoplasmas in declining pears in Southern Australia. Plant Dis. 81, 254–258. 10.1094/PDIS.1997.81.3.254 30861766

[B66] Schneider B. Padovan A. Rue S. Eichner R. Davis R. Bernuetz A. (1999). Detection and differentiation of phytoplasmas in Australia: an update. Aust. J. Agric. Res. 50, 333–342. 10.1071/A98106

[B67] Smith G. R. Braithwaite K. S. Cronjé C. P. R. (2001). The viral and phytoplasma forms of yellow leaf syndrome of sugarcane, in Proceedings International Society Sugar Cane Technologists, ed Hogarth D. M.(Brisbane, QLD: International Society Of Sugar Cane Technologists), 614–617.

[B68] Streten C. Conde B. Herrington M. Moulder J. Gibb K. (2005). *Candidatus* Phytoplasma australiense is associated with pumpkin yellow leaf curl disease in Queensland, Western Australia and the Northern Territory. Australasian Plant Pathol. 34, 103–105. 10.1071/AP04077

[B69] Streten C. Gibb K. S. (2006). Phytoplasma diseases in sub-tropical and tropical Australia. Australas. Plant Pathol. 35, 129–146. 10.1071/AP06004

[B70] Tairo F. Jones R. A. C. Valkonen J. P. T. (2006). Phytoplasma from little leaf disease affected sweetpotato in Western Australia: detection and phylogeny. Ann. Appl. Biol. 149, 9–14. 10.1111/j.1744-7348.2006.00065.x

[B71] Tanne E. Boudon-Padieu E. Clair D. Davidovich M. Melamed S. Klein M. (2001). Detection of phytoplasma by polymerase chain reaction of insect feeding medium and its use in determining vectoring ability. Phytopathology 91, 741–746. 10.1094/PHYTO.2001.91.8.741 18944030

[B72] Tran-Nguyen L. Blanche K. R. Egan B. Gibb K. S. (2000). Diversity of phytoplasmas in northern Australian sugarcane and other grasses. Plant Pathol. 49, 666–679. 10.1046/j.1365-3059.2000.00498.x

[B73] Tran-Nguyen L. T. T. Kube M. Schneider B. Reinhardt R. Gibb K. S. (2008). Comparative genome analysis of “*Candidatus* Phytoplasma australiense” (subgroup *tuf*-Australia I; *rp-A*) and “*Ca*. Phytoplasma asteris” strains OY-M and AY-WB. J. Bacteriol. 190, 3979–3991. 10.1128/JB.01301-07 18359806 PMC2395047

[B74] Tran-Nguyen L. T. T. Persley D. M. Gibb K. S. (2003). First report of phytoplasma disease in capsicum, celery and chicory in Queensland, Australia. Australas. Plant Pathol. 32, 559–560. 10.1071/AP03055

[B75] Tran-Nguyen L. T. T. Smith S. H. Liberato J. R. (2012). Sweet potato little leaf strain V4 phytoplasma associated with snake bean in the Northern Territory, Australia. Australas. Plant Dis. Notes 7, 147–150. 10.1007/s13314-012-0071-9

[B76] Veerakone S. Tang J. Z. Ward L. I. Liefting L. W. Perez-Egusquiza Z. Lebas B. S. M. (2015). A review of the plant virus, viroid, liberibacter and phytoplasma records for New Zealand. Australas. Plant Pathol. 44, 463–514. 10.1007/s13313-015-0366-3

[B77] Vega F. E. Davis R. E. Barbosa P. Dally E. L. Purcell A. H. Lee I. (1993). Detection of a plant pathogen in a nonvector insect species by the polymerase chain reaction. Phytopathology 83, 621–624. 10.1094/Phyto-83-621

[B78] Wang Q. Guo Y. Wang N. Li Y. Chen W. Chen W. (2014). Identification of a conserved core genome with group-specific genes from comparative genomics of ten different *Candidatus* Phytoplasma strains. J. Phytopathol. 162, 650–659. 10.1111/jph.12239

[B79] Weintraub P. G. Beanland L. (2006). Insect vectors of phytoplasmas. Annu. Rev. Entomol. 51, 91–111. 10.1146/annurev.ento.51.110104.151039 16332205

[B80] White D. T. Blackall L. L. Scott P. T. Walsh K. B. (1998). Phylogenetic positions of phytoplasmas associated with dieback, yellow crinkle and mosaic diseases of papaya, and their proposed inclusion in “*Candidatus* Phytoplasma australiense” and a new taxon, “*Candidatus* Phytoplasma australasia.” Int. J. Syst. Evol. Microbiol. 48, 941–951. 10.1099/00207713-48-3-941 9734050

[B81] Wilson D. Blanche K. R. Gibb K. S. (2001). Phytoplasmas and disease symptoms of crops and weeds in the semi-arid tropics of the Northern Territory, Australia. Australas. Plant Pathol. 30, 159–163. 10.1071/AP01015

[B82] Yang S. Habili N. Aoda A. Dundas I. Paull J. Randles J. (2013). Three group 16SrII phytoplasma variants detected in co-located pigeonpea, lucerne and tree medic in South Australia. Australas. Plant Dis. Notes 8, 125–129. 10.1007/s13314-013-0113-y

[B83] Zhao Y. Davis R. E. (2016). Criteria for phytoplasma 16Sr group/subgroup delineation and the need of a platform for proper registration of new groups and subgroups. Int. J. Syst. Evol. Microbiol. 66, 2121–2123. 10.1099/ijsem.0.000999 26928977

