# Corrigendum: Phytoplasmas—The “Crouching Tiger” Threat of Australian Plant Pathology

**DOI:** 10.3389/fpls.2018.01298

**Published:** 2018-10-26

**Authors:** Jian Liu, David Gopurenko, Murray J. Fletcher, Anne C. Johnson, Geoff M. Gurr

**Affiliations:** ^1^State Key Laboratory of Ecological Pest Control for Fujian and Taiwan Crops, Fujian Agriculture and Forestry University, Fuzhou, China; ^2^Institute of Applied Ecology, Fujian Agriculture & Forestry University, Fuzhou, China; ^3^Graham Centre for Agricultural Innovation (Charles Sturt University & NSW Department of Primary Industries), Orange, NSW, Australia; ^4^NSW Department of Primary Industries, Wagga Wagga Agricultural Institute, Wagga Wagga, NSW, Australia

**Keywords:** “*Candidatus* Phytoplasma”, 16S rRNA, biosecurity, taxonomy, biodiversity, vector, seed transmission, host range

In the original article, information for phytoplasmas in Table 1 did not fully reflect recent changes in taxonomy, or showed changes only as footnotes. Corrections have been made in the sections below and in Table 1.

**Table 1:** Taxonomic and biological information on phytoplasmas in Australia (empty cells denote the absence of available information).

**16Sr group**	***“Candidatus* Phytoplasma” name**	**Phytoplasma trivial name**	**Host plant species**	**Potential vectors**	**Location^+^**	References^*^
II	australasiae^a^	Australian lucerne yellows	*Medicago sativa, Carica papaya*	*Orosius argentatus, Austroagallia torrida, Orosius* spp., *Batracomorphus* sp.	South Australia, New South Wales, Northern Territory	Padovan and Gibb, 2001^*^; Pilkington et al., 2003^*^; Yang et al., 2013
II		Bonamia pannosa little leaf	*Bonamia pannosa*		Northern Territory	Schneider et al., 1999; Padovan and Gibb, 2001
II		Cactus witches' broom	*Carica papaya*		Northern Territory	Padovan and Gibb, 2001
II		Cocky apple witches' broom	*Planchonia careya*		Queensland	Davis et al., 2001
II		Waltheria little leaf	*Mitracarpus hirtus, Saccharum* sp*., Spermacocci sp., Waltheria indica, Carica papaya*		Northern Territory	Schneider et al., 1999; Tran-Nguyen et al., 2000; Padovan and Gibb, 2001; Wilson et al., 2001
II	australasiae	Tomato big bud	*Achyranthes aspera, Aeschynomene* spp.*, Alysicarpus rugosus, Amaranthus* sp., *Apium graveolens, Arachis* spp.*, Boeharvia* sp., *Brugmansia x candida, Capsicum annuum, Carica papaya, Catharanthus roseus, Cajanus cajan, Citrus paradisi, Crotalaria* spp., *Cenchrus ciliaris, Cichorium intybus, Cleome viscosa, Cucurbita maxima, Cynodon dactylon, Daucus carota, Emilia sonchifolia, Eragrostis falcata, Eriachne obtusa, Euphorbia milii, Evolvulus* sp., *Gerbera* sp., *Goodenia* sp., *Guizotia abyssinica, Ipomoea* spp.*, Lactuca sativa, Lycopersicon esculentum, Macroptilium* spp., *Medicago sativa, Mucuna pruriens, Passiflora* sp., *Phlox* sp., *Physalis minima, Ptilotus distans, Rhynchosia minima, Saccharum* sp., *Sarcochilus hartmanii × S. falcatus, Sesamum indicum, Sida cordifolia, Lycopersicon esculentum, Solanum melongena, Stylosanthes scabra, Trifolium repens, Vigna* spp.*, Vitis vinifera, Zinnia elegans*,	*Austroagallia torrida*	Northern Territory, New South Wales, Queensland, Western Australia, Victoria	Gibb et al., 1995; Davis et al., 1997b; Gowanlock et al., 1998; De La Rue et al., 1999; Tran-Nguyen et al., 2000, 2003; Wilson et al., 2001^*^; Pilkington et al., 2004; Streten and Gibb, 2006
II	aurantifolia	Chickpea little leaf	*Cicer arietinum*		Western Australia	Saqib et al., 2005
II	australasiae	Papaya yellow crinkle	*Carica papaya*		Queensland	Gibb et al., 1996; White et al., 1998
II	australasiae	Papaya mosaic	*Carica papaya*		Queensland	Gibb et al., 1996; White et al., 1998
II		Tree medic witches' broom	*Medicago arborea*		South Australia	Yang et al., 2013
II		Pigeonpea phyllody	*Cajanus cajan*		South Australia	Yang et al., 2013
II		Pigeon pea little leaf	*Arachis* spp., *Catharanthus roseus, Crotalaria* spp.*, Desmodium triflorum, Indigofera* sp., *Macroptilium bracteatum Pterocaulon* sp. *Sesuvium portulacastrum, Stylosanthes* spp.*, Vigna radiata*		Northern Territory, Queensland, Torres Strait	Schneider et al., 1999; De La Rue et al., 2001; Padovan and Gibb, 2001; Wilson et al., 2001; Davis et al., 2003; Streten and Gibb, 2006
II-D	australasiae	Pale purple coneflower witches' broom	*Echinacea pallida*		Tasmania	Pearce et al., 2011
II-D	australasiae	Sweet potato little leaf	*Alysicarpus vaginalis, Aphyllodium* sp., *Arachis* spp., *Cajanus marmoratus, Carica papaya, Catharanthus roseus, Centrosema pascuorum, Citrus* sp., *Cleome viscosa, Crotalaria* spp., *Cucurbita maxima, Cyanthillium* spp., *Desmodium* spp., *Emilia sonchifolia, Indigofera* spp., *Ipomoea batatas, Macroptilium gracile, Medicago sativa, Mitracarpus hirtus, Nicotiana tabacum, Pachyrhizus erosus, Physalis minima, Rhynchosia minima, Senna obtusifolia, Sesamum indicum, Stylosanthes* ssp., *Tridax procumbens, Vigna* spp.	*Austroagallia torrida, Orosius* spp., *Batracomorphus* sp.	Torres Strait, Northern Territory, Western Australia, New South Wales	Gibb et al., 1995^*^; Liu et al., 1996; Davis et al., 1997b; Schneider and Gibb, 1997^*^; De La Rue et al., 1999, 2001; Padovan and Gibb, 2001; Wilson et al., 2001; Davis et al., 2003; Streten and Gibb, 2006; Tairo et al., 2006; Tran-Nguyen et al., 2012
XI-B		Cynodon white leaf	*Cynodon dactylon, Dactyloctenium aegyptium*		Northern Territory, Western Australia	Schneider et al., 1999; Tran-Nguyen et al., 2000; Blanche et al., 2003
XI-B		Sorghum grassy shoot	*Dactyloctenium* spp.*, Sorghum stipoideum, Whiteochloa* spp., *Chloris inflata, Whiteochloa cymbiformis*		Western Australia, Northern Territory	Tran-Nguyen et al., 2000; Blanche et al., 2003
XII		Australian lucerne yellows	*Medicago sativa*		New South Wales	Getachew et al., 2007
XII		Papaya dieback	*Carica papaya*		Queensland	Gibb et al., 1996; White et al., 1998
XII-B	australiense	Pumpkin yellow leaf curl	*Cucurbita maxima, C. moschata*		Queensland, Western Australia, Northern Territory	Streten et al., 2005
XII-B	australiense	Cenchrus bunchy shoot	*Cenchrus setiger*		Western Australia	Tran-Nguyen et al., 2000
XII-B	australiense	Strawberry green petal disease	*Fragaria* x *ananassa*		Queensland	Padovan et al., 2000
XII-B	australiense	Strawberry lethal yellows	*Fragaria* x *ananassa*		Queensland	Padovan et al., 2000
XII-B	australiense	Australian grapevine yellows^b^	*Vitis vinifera, Carica papaya*		South Australia, Queensland	Davis et al., 1997a,b; Davis and Sinclair, 1998; Davis et al., 2003
XXIII^c^		Buckland Valley grapevine yellows	*Vitis vinifera*		Victoria	Constable et al., 2003; Streten and Gibb, 2006; Zhao and Davis, 2016
XXV^d^		Weeping tea tree witches' broom	*Melaleuca* spp.		Queensland	Davis et al., 2003; Zhao and Davis, 2016
XXXIII		Allocasuarina yellows	*Allocasuarina muelleriana*		South Australia	Gibb et al., 2003; Zhao and Davis, 2016
		Poinsettia branching^e^	*Euphorbia pulcherrima*			Schneider et al., 1999
		Galactia little leaf	*Galactia tenuiflora*		Northern Territory	Schneider et al., 1999; Padovan and Gibb, 2001
		Sorghum bunchy shoot	*Sorghum stipoideum*			Tran-Nguyen et al., 2000
		Stylosanthes little leaf	*Arachis pintoi, Carica papaya, Saccharum* sp*., Sesuvium portulacastrum, Stylosanthes scabra*	*Austroagallia torrida, Orosius* spp., *Batracomorphus* sp.	Northern Territory, Queensland, New South Wales	Schneider et al., 1999; Tran-Nguyen et al., 2000; De La Rue et al., 2001; Padovan and Gibb, 2001; Davis et al., 2003; Gopurenko et al., 2016
		Sugarcane white leaf	*Saccharum* sp.		Western Australia, Queensland	Tran-Nguyen et al., 2000
		Vigna little leaf	*Vigna lanceolata, Carica papaya, Tridax procumbens*	*Austroagallia torrida, Batracomorphus* sp.	Northern Australia	Schneider et al., 1999; De La Rue et al., 2001; Padovan and Gibb, 2001
		Mundulla yellows disease^f^	*Eucalyptus camaldulensis, E. baxteri, E. leucoxylon*		South Australia	Hanold et al., 2006
		Paulownia witches' broom^g^	*Paulownia* sp.		Western Australia	Bayliss et al., 2005

* *Denotes reference for vector data*.

+ *Location data are from the listed references but not every plant species was diseased in every location*.

a *A new taxon, Ca. Phytoplasma australasia was proposed (White et al., 1998) to include the phytoplasma associated with papaya yellow crinkle and papaya mosaic (as well as tomato big bud) but later revised to “Ca. australasiae” (to include the papaya-associated phytoplasmas but not TBB; Firrao et al., 2005)*.

b *Davis and Sinclair (1998) moved the AGY phytoplasma from the 16SrI group into the stolbur group (16SrXII) and designated it subgroup B*.

c *Constable et al. (2003) reported a close relationship to 16Sr I. Zhao and Davis (2016) subsequently placed this into a new group: 16SrXXIII*.

d *Zhao and Davis (2016) placed this into this new group and potentially a new “Ca. Phytoplasma” species*.

e *This phytoplasma has not been found in economically important field crops*.

f *Tentative data only for a phytoplasma etiology*.

g RFLP patterns showed high similarity to “Candidatus Phytoplasma australiense.”

## Abstract

Phytoplasmas are insect-vectored bacteria that cause disease in a wide range of plant species. The increasing availability of molecular DNA analyses, expertise, and additional methods in recent years has led to a proliferation of discoveries of phytoplasma-plant host associations and in the numbers of taxonomic groupings for phytoplasmas. The widespread use of common names based on the diseases with which they are associated, as well as separate phenetic and taxonomic systems for classifying phytoplasmas based on variation at the 16S rRNA-encoding gene, complicates interpretation of the literature. We explore this issue and related trends through a focus on Australian pathosystems, providing the first comprehensive compilation of information for this continent, covering the phytoplasmas, host plants, vectors, and diseases. Of the 33 16Sr groups reported internationally, only groups II, XI, XII, XXIII, XXV, and XXXIII have been recorded in Australia and this highlights the need for ongoing biosecurity measures to prevent the introduction of additional pathogen groups. Many of the phytoplasmas reported in Australia have not been sufficiently well-studied to assign them to 16Sr groups so it is likely that unrecognized groups and sub-groups are present. Wide host plant ranges are apparent among well studied phytoplasmas, with multiple crop and non-crop species infected by some. Disease management is further complicated by the fact that putative vectors have been identified for few phytoplasmas, especially in Australia. Despite rapid progress in recent years using molecular approaches, phytoplasmas remain the least well-studied group of plant pathogens, making them a “crouching tiger” disease threat.

## Issue 2: Complex taxonomic nomenclature, paragraphs 2 and 3

Second, as molecular methods became available, workers were able to group and phenetically classify phytoplasmas using restricted fragment length polymorphism (RFLP) analysis of a PCR amplified portion of the 16S rRNA gene with a defined set of restriction enzymes (Lee et al., 1998). The RFLP profiles generated for different phytoplasmas are generally consistent with sequence-based phylogenetic analyses of the 16S rRNA gene, particularly in the co-identification and grouping of related strains. The 33 16Sr groups currently defined each have a similarity of less than 85% compared with any representative phytoplasma from within an established 16Sr group (Zhao and Davis, 2016). Table 1 summarizes available information on the 16Sr groups reported in Australian studies. Of the 33 16Sr groups reported internationally, only groups II, XI, XII, XXIII, XXV, and XXXIII have been recorded in Australia and this highlights the need for ongoing biosecurity measures to prevent the introduction of additional pathogen groups.

Third, phytoplasmas are classified in the provisional genus “*Candidatus* Phytoplasma” (IRPCM, 2004). To date, there are 42 formally described species and ten potentially novel phytoplasma species (Davis et al., 2015). This number exceeds the current number of 16s rRNA groups because some of these groups contain several “*Candidatus* Phytoplasma” species. At least 100 subgroups are known (Dickinson and Hodgetts, 2013). According to Phytoplasma/Spiroplasma Working Team-Phytoplasma Taxonomy Group, a novel “*Ca*. Phytoplasma” species description should refer to a single, unique 16S rRNA gene sequence (>1,200 bp), and a strain can be recognized as a novel “*Ca*. Phytoplasma” species if its 16S rRNA gene sequence has <97.5% similarity to that of any previously described “*Ca*. Phytoplasma” species (Duduk and Bertaccini, 2011). Additional biological characters such as antibody specificity, host range and vector transmission specificity as well as genetic markers can also be used in an integrative taxonomy approach for species differentiation. Of the 42 recognized “*Ca*. Phytoplasma” species, only *Ca*. Phytoplasma aurantifolia, *Ca*. Phytoplasma australasiae and *Ca*. Phytoplasma australiense are reported in Australia (Table 1) but uncertainty exists because many papers appear without *Ca*. Phytoplasma names which are used consistently only in the case of the GenBank database.

The authors apologize for this error and state that this does not change the scientific conclusions of the article in any way. The original article has been updated.

### Conflict of interest statement

The authors declare that the research was conducted in the absence of any commercial or financial relationships that could be construed as a potential conflict of interest.
